# Assessing Classic Maya multi-scalar household inequality in southern Belize

**DOI:** 10.1371/journal.pone.0248169

**Published:** 2021-03-24

**Authors:** Amy E. Thompson, Gary M. Feinman, Keith M. Prufer

**Affiliations:** 1 Negaunee Integrative Research Center, The Field Museum of Natural History, Chicago, Illinois, United States of America; 2 Department of Geography and the Environment, University of Texas at Austin, Austin, Texas, United States of America; 3 Department of Anthropology, University of New Mexico, Albuquerque, New Mexico, United States of America; Utah State University, UNITED STATES

## Abstract

Inequality is present to varying degrees in all human societies, pre-modern and contemporary. For archaeological contexts, variation in house size reflects differences in labor investments and serves as a robust means to assess wealth across populations small and large. The Gini coefficient, which measures the degree of concentration in the distribution of units within a population, has been employed as a standardized metric to evaluate the extent of inequality. Here, we employ Gini coefficients to assess wealth inequality at four nested socio-spatial scales–the micro-region, the polity, the district, and the neighborhood–at two medium size, peripheral Classic Maya polities located in southern Belize. We then compare our findings to Gini coefficients for other Classic Maya polities in the Maya heartland and to contemporaneous polities across Mesoamerica. We see the patterning of wealth inequality across the polities as a consequence of variable access to networks of exchange. Different forms of governance played a role in the degree of wealth inequality in Mesoamerica. More autocratic Classic Maya polities, where principals exercised degrees of control over exclusionary exchange networks, maintained high degrees of wealth inequality compared to most other Mesoamerican states, which generally are characterized by more collective forms of governance. We examine how household wealth inequality was reproduced at peripheral Classic Maya polities, and illustrate that economic inequity trickled down to local socio-spatial units in this prehispanic context.

## Introduction

Inequality is universal in human societies but its degree and the ways it manifests vary across time, space, and the scale of interpersonal networks and institutions [1, 2]. Even in single regional and societal settings, degrees of inequity are highly variable with changing circumstances and contexts [3–6, see also 7]. Here, using the Gini coefficient as a standardized measure of inequality and applied to variation in house sizes as an indicator of wealth differences, we demonstrate that high degrees of wealth inequality were present in peripheral Classic Maya polities, where it was manifested at multiple, nested scales from the micro-region to small, neighborhood units.

Based on the measurements of wealth inequality at Uxbenká and Ix Kuku’il, two Classic Maya centers in the southeastern periphery of the Classic Maya (250–900 CE) region, and by situating these findings in broader comparative contexts, we illustrate that degrees of wealth inequality in this region were comparable to those in the Classic Maya heartland. We show that compared to other Mesoamerican centers, wealth inequality amongst the Classic Maya was elevated [4]. We propose that these patterns of economic inequity reflect to a degree the importance of elite socioeconomic networks, which served as a basis of wealth and power for Classic Maya rulers. We document that access to trade and interpersonal networks fostered wealth inequality even at the edges of the Classic Maya world within nested scales of community.

Our analyses, focused on Uxbenká and Ix Kuku’il, are undergirded by a broader theoretical framework that has established a relationship between degrees of inequality and modes of governance [3, e.g., 8, 9]. At Classic Maya polities, elite networks [10] facilitated the movement of luxury goods through a range of exchange networks [11–13]. The control of goods that were transferred through these networks fostered the maintenance of power by autocratic rulers [e.g., 14]. These networks extended down to nobles (e.g., *sajal* [15, 16]) in districts and to neighborhood heads [17] through inter- and intra-polity economic relationships [18]. These patterns contrast with degrees of wealth inequality and governance documented in the highlands of prehispanic Mesoamerica during the Classic period, including Central Mexico and Oaxaca, where governance was more collective and financed to a greater degree from local sources [9].

Specifically, in this investigation, we examine differences in house sizes at multiple scales for the settlements of Uxbenká and Ix Kuku’il. Through these analyses, we not only illustrate that wealth differentials were amply expressed even at the southeastern limits of the Classic Maya world, but that these inequities were manifest and trickled down to more localized spatial scales even among low-density settlement systems. Although implemented for a specific archaeological case, our findings are potentially more broadly relevant as key relations that we report also have been recognized in a suite of comparative contexts across different periods and time scales [19–22].

### Autocratic and collective forms of governance

Before examining wealth inequality at Uxbenká and Ix Kuku’il, we briefly outline our theoretical foundations and a number of key analytical presumptions and procedures. In large human cooperative arrangements, modes of governance can be compared along a continuous dimension [23, 24]. At one end of the continuum, power is concentrated, often in a single individual, kin group, or a small cabal, while at the other extreme, power is distributed, and governance is more collectively implemented. Drawing on the research of Levi [25] and Blanton and Fargher [8, 19], we see the financing of governance and rule, whether revenues were derived from largely internal (from the local population) or external (from foreign or easily monopolized sources) resources, as a key factor underpinning the relative concentration of power. Variability in revenue source or fiscal financing impacts the levels of accountability that principals, or leaders, have in relation to subordinates, and the likelihood that governance and power will be shared or highly concentrated [8, 26].

Blanton and Fargher [8, 19] build on the comparative study of Levi [25] to propose that more representative or collective forms of leadership are found where those with power are more directly dependent on the local populace for their economic underpinnings. Alternatively, exclusionary/autocratic rule is more apt to occur where principals are less dependent on their immediate populace for economic support. Rather, principals in autocratic regimes acquire their funds of power from external sources, which include the monopolization of exchange routes and the control of luxury goods [19]. When reliant on internal revenues, principals are more prone to cede voice or provide concessions in the form of public goods, representation, or services to curtail subordinate resistance or lessen outmigration [27]. In exclusionary regimes, principals are less dependent on the extraction of resources from their immediate populace and can, therefore, afford diminished representation, voice, or public goods and services. In this sense they are freer to exercise more personalized power and interactions between principals and subalterns may be more transactional along the lines of patron-client like relationships.

### Measuring wealth inequality

To probe relationships between governance and differences in wealth, we employ the Gini coefficient to assess variation in house sizes for a suite of spatial units. Recently, among modern communities patterns of wealth inequality have been linked to the intergenerational transmission of wealth [2]. Wealth inequality also was associated with forms of governance among large, cross-cultural archaeological samples from the old and new world [28]. However, most archaeological assessments of wealth inequality have been implemented at the level of large settlements or polities [e.g., 4–6]. This study is one of the first to trace inequality through multiple, nested scales within a micro-region (for other recent examples see [29–31]).

To assess wealth inequality we examine house size, a frequently employed measure of wealth in archaeological contexts [3, 5, 30, 32–39]. Although we recognize that some may prefer a multidimensional approach that includes the analysis of artifact assemblages or storage space [29, 40, 41], such considerations either were not possible in our study context or would have required a marked diminishment in the size of our analytical sample. The use of house size as a measure of wealth provides the opportunity to examine large archaeological samples that are sufficiently robust for quantification. Our reliance on pedestrian surveys allows us to avoid potential biases present in excavation-based samples, which generally are small. Likewise, house size “provides an easily comparable unit of measurement, thus allowing cross-cultural analysis” [33 p2].

House size has been used to assess wealth inequality in archaeological contexts in the Maya region [4, 30, 34, 35, 37, 42, 43], other areas of prehispanic Mesoamerica [3, 39], and more geographically dispersed regions including the NW Coast of North America [32, 41], the US SW [31, 44–48], the Mississippian heartland [49], Colombia [40], Europe [5, 50], and Eurasia [33, 50, 51]. No other measure allows for regional and global cross-cultural comparisons of wealth inequality using a consistent metric: house size. As illustrated through prior comparative investigations [5, 28, 33], the extent of the wealth inequality between different samples of domestic units can be effectively assessed using the Gini coefficient, thereby providing a foundation for geographically-wide, cross-temporal comparisons [52, 53]. We adhere to the assumption that house size is an appropriate proxy for household wealth, although, when possible, we supplement this metric with qualitative discussions of other indicators of wealth inequality.

The Gini coefficient “measures the degree of concentration of a quantity among the units of a population” [38 p24–25] and can serve to produce a single metric for the degree of inequality [54]. Gini coefficients closer to 0 represent greater equality (less inequality) within the population, while Gini coefficients closer to 1 represent greater inequality (less equality) [22]. We use the Gini as a standardized method to evaluate wealth inequality based on the distribution and dispersion of measures of wealth, in this case, house size. As the Gini coefficient yields a single measurement, based on the distribution of a given sample [55], its use facilitates comparisons across different spatiotemporal contexts. The Gini coefficient is visually complemented with the Lorenz curve, a graphical distribution of wealth wherein larger areas under the curve represent less equally distributed populations and smaller areas under the curve represent more equally distributed populations [35].

### Archaeological focus and background

We assess degrees of wealth inequality between Classic Maya polities, analyzing a micro-region, the Rio Blanco valley, in which two neighboring polities, Uxbenká and Ix Kuku’il, were situated. Although interconnected with the larger Classic Maya world, these two rural polities in the Rio Blanco watershed of southern Belize nonetheless were spatially removed from larger and more populous polities, such as Tikal, Calakmul, and Caracol, in the heartland of the Maya region [10]. Although Uxbenká, Ix Kuku’il, and other polities in southern Belize, are located 150 kilometers from the Classic Maya heartland of the central Petén, they were unambiguously connected to the political economies of more central regions and polities, such as Tikal and El Perú in the Central Petén, Cahal Pech in western Belize, and Copan and Quirigua in the southeastern periphery [56–59].

#### Southern Belize: The Rio Blanco valley

**Fig 1:**
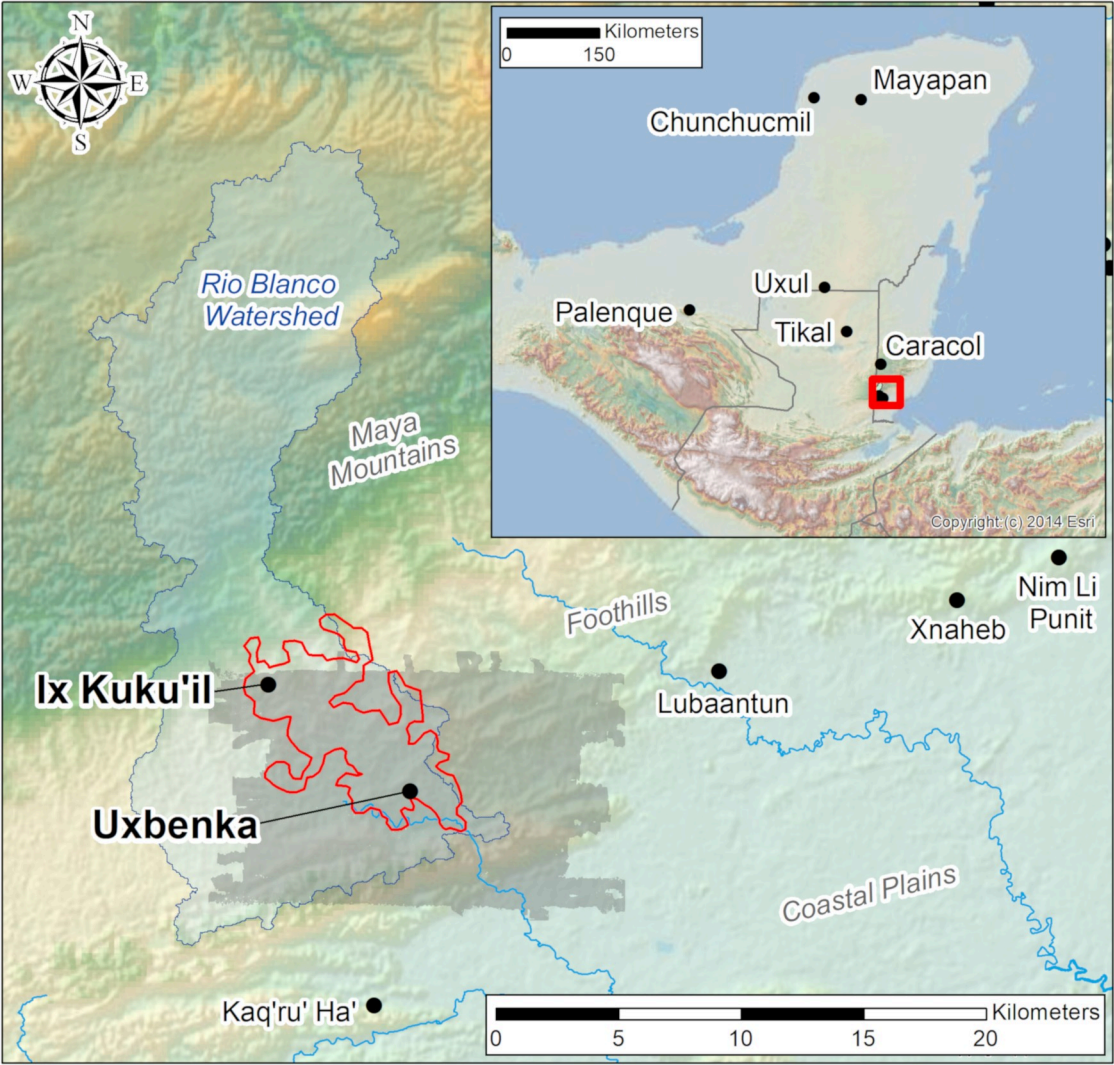
Map of the study region. Map with the lidar zone (shaded grey) and Uxbenká and Ix Kuku’il pedestrian survey zone highlighted (red outline) within the Rio Blanco valley (larger watershed is shaded blue with blue outline). Maya polities with published Gini coefficients for plazuelas/houselots are labeled on the inset map of the Maya region. Base map images are the intellectual property of Esri and are used herein under license. Copyright 2014 Esri and its licensors. All rights reserved.

Uxbenká and Ix Kuku’il are situated in the fertile, hilly uplands of the Rio Blanco valley (Fig 1). Dispersed across several river valleys and drainages in southern Belize, seven Classic Maya polities are located in these uplands, a highly productive agricultural landscape [60]. Using traditional, non-mechanized methods and without chemical fertilizers, farmers today plant corn, beans, and squash for the home and market [61]. Cacao is grown by hundreds of small-holder farmers with surplus going exclusively to the export market [62]. Historic documents from the 16^th^ century describe economically important cacao cultivation in southern Belize [63]. Dried cacao seeds were still exchanged commercially and were an item of great symbolic value in life cycle events among Maya communities into the 21^st^ century [64]. The highly productive agricultural soils in the Rio Blanco valley have long supported cacao orchards [65] and likely attracted early settlers to the region.

Southern Belize polities are characterized by relatively high numbers of elaborate stelae depicting political leaders, a general lack of masonry superstructures [58], and dispersed, low-density settlements [66, 67]. Residential plazuelas (henceforth referred to simply as plazuelas), are composed of domestic structures situated around a central plaza [68]. Generally, these are situated on discrete modified hilltops and they represent a single household, or domestic unit [69, 70]. The settlement systems of Uxbenká and Ix Kuku’il together contain hundreds of plazuelas that are dispersed across the landscape and cluster into neighborhoods and districts [71–73]. Many of these plazuelas are small with low household structures less than 50 cm high [67] and are often neglected in archaeological studies of settlements that focus on larger buildings with more abundant remains of cultural materials. Neighborhoods are defined as social units that are spatially discrete where social and economic interactions occurred between residents on a daily basis [74, 75]. Districts are composed of a number of adjacent neighborhoods and include high-status residences and administrative or public architecture [76]. At Uxbenká and Ix Kuku’il, neighborhoods and districts are composed of dispersed residential units of varying size, a pattern noted in communities with autocratic forms of governance [77]. In conjunction with the Rio Blanco valley micro-region and the two polities, neighborhoods and districts were our analytical units, which allowed us to compare wealth inequality at nested scales.

In addition to their generally similar settlement patterns, Uxbenká and Ix Kuku’il had relatively similar developmental histories. The earliest permanent structures were built during the Late Formative (300 BCE– 250 CE). Growth occurred during the Early Classic (250–600 CE) and Late Classic (600–800 CE), which was followed by population decline during the Terminal Classic (800–1000 CE) [72]. Small occupations were maintained throughout the Terminal Classic and into the Early Postclassic (1000–1250 CE) [60]. However, many of the households at Uxbenká were established earlier, by 400 CE, compared to the households at Ix Kuku’il, which were occupied later, after 600 CE [78, 79].

The occupational histories, sizes and distributions of houses, and the definitions of districts and neighborhoods are based on archaeological investigations conducted between 2005 and 2018. Uxbenká has been the subject of a decade of investigations while five years of research were conducted at Ix Kuku’il. The research design at both sites focused on extensive settlement survey and excavations in large and small households alike to gain a holistic understanding of the Classic Maya in the Rio Blanco valley.

#### Uxbenká

**Fig 2:**
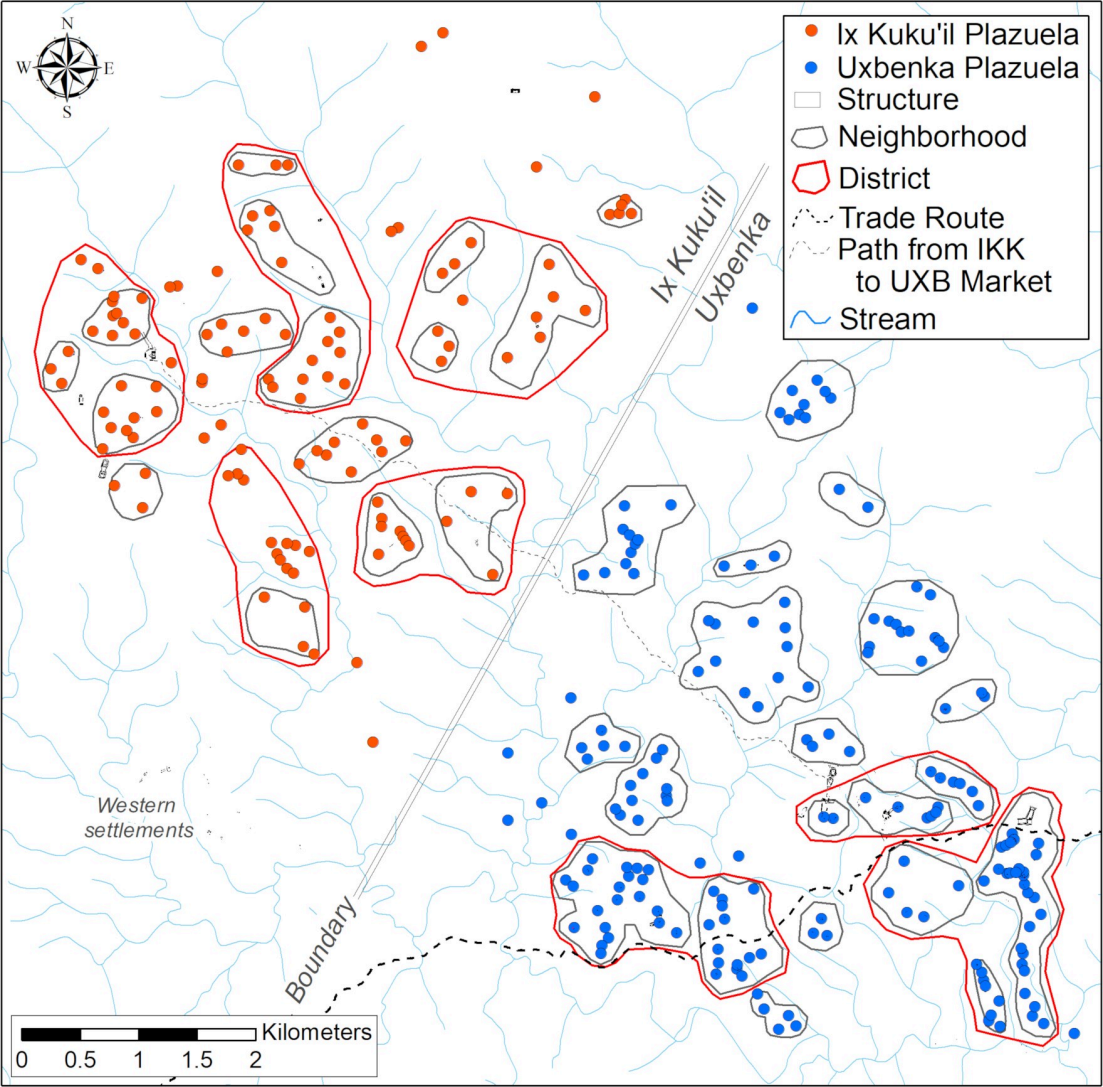
Settlement map of Uxbenká and Ix Kuku’il. Plazuela, neighborhood, district, and polity boundaries of Uxbenká and Ix Kuku’il. Trade routes based on Least Cost Path analyses and perennial streams are also displayed on the map.

Uxbenká is composed of 10 groups of monumental civic architecture that are surrounded by 180 plazuelas. The plazuelas are clustered into 20 neighborhoods and the monumental architecture is dispersed into three districts. The districts are situated along an east-west transportation corridor (Fig 2) approximately 1.5 km apart. This transportation corridor, henceforth trade route, was previously modeled using a Least Cost Path (LCP) analysis [see 72 S2 Text, 80]. Land near the trade route was preferentially selected for settlement by the early occupants of Uxbenká. The trade route connected Uxbenká to the Gulf of Honduras with marine resources and coastal trade networks as well as large consumer populations in the central Petén. Uxbenká’s position along the trade route allowed its principals to mediate long-distance trade [72].

Districts include neighborhoods and plazuelas, so that residential configurations are nested at multiple scales. Each district has a District Seat, which houses large, noble residences that are situated near monumental architectural features [72] and surrounded by smaller households. The District 1 Seat is Group L, which is attached via a short causeway to a ceremonial plaza (Group A) that contains 23 stelae and a northern triadic pyramid. Group L was constructed around 300 CE and includes several royal tombs. The District 2 Seat is Settlement Group 25, which is a multi-plazuela household group with a temple and several tombs. The largest temple (and tomb) is in Plazuela 25E (P 25E) which was also founded around 300 CE [81]. The District 3 Seat is Group I, which was founded shortly before 300 CE. Group I contains residential plazuelas with multiple elaborate tombs attached to a large, public plaza with a temple and a ball court.

#### Ix Kuku’il

Ix Kuku’il lies northwest of Uxbenká. The Rio Blanco river valley, which has few small and sparsely distributed plazuelas on either bank, likely served as a boundary between the two polities and their settlement systems. Ix Kuku’il has 10 decentralized areas of civic-ceremonial architecture dispersed across the landscape surrounded by 122 residential plazuelas. Ix Kuku’il is further divided into five districts and 16 neighborhoods (Fig 2). The Seat of District 1 is Plazuela 51A (P 51A), which is a high-status household located near Group A. Group A, likely the focal point for polity leadership, contains a 10 m tall inline eastern triadic temple and a massive 4.2 m tall uncarved stela [82]. District 2 has two minor temples located in elite residential plazuelas P 19B and P 127C. The District 3 Seat is P 32A, which is an elite household with a 5 m tall temple, similar to P 25E at Uxbenká. P 32A is near a hilltop shrine (Group E) and the only identified ballcourt at Ix Kuku’il (Group F). District 4 contains a minor temple located in Group K and a small, northern inline triadic temple in P 90. Finally, District 5 contains a hilltop shrine in Group I and a temple in Group J. A LCP was modeled between Ix Kuku’il Group A and the Uxbenka market at Group D. This route represents the path of least resistance across the landscape. At present, the chronology for Ix Kuku’il is less precisely defined than for Uxbenká. Most of Ix Kuku’il’s plazuelas were founded during the Late Classic, but several of the elite residential plazuelas, including P 32A, P 19B, and P 51A, were founded during the Early Classic II (400–600 CE) [78].

## Materials and methods

All research at Uxbenká and Ix Kuku’il was conducted under permits (to K.M.P.) authorized by the Belize Institute of Archaeology to the Uxbenká Archaeological Project. Fieldwork was conducted after consultation and permissions were granted by the leadership of the communities of Santa Cruz and San Jose, on whose lands the archaeological sites are located. Consistent with professional ethical obligations, public presentations of research results to the communities were done on an annual basis, and copies of published reports and other products were provided to community leaders for inclusion in primary school libraries.

To assess wealth inequality at Uxbenká and Ix Kuku’il, we measured (a) basal area (m^2^), (b) surface area (m^2^), and (c) volume (m^3^) for each plazuela including structure platforms, plazas, and spaces between structures. The data are 302 plazuelas across an area of 42 km^2^. These are further divided into a total of eight districts and 36 unique neighborhoods, allowing us to evaluate wealth inequality at multiple, nested scales in a single community, the micro-region of the Rio Blanco Valley.

### Units of analysis: Plazuelas

#### House size

In the Maya area, household wealth inequality is assessed through differences in the size of construction, types of materials, and architectural elaborations as proxies for labor investment [44, 83]. In southern Belize, plazuela hilltops were flattened and slopes were raised using boulders and crushed fill to create larger footprints. The raised surfaces were covered by smaller boulders, then cobbles, and then covered with plaster or mud to create living surfaces. Landscape modifications are more pronounced at larger plazuelas compared to smaller plazuelas. For example, at Uxbenká’s P 25E, the hilltop area was expanded more than 50% based on excavation and Light Detection and Ranging (lidar) data [84 Table 2]. Such hilltop expansions would have required significant labor investment.

Across the Maya region, elite houses may contain intricate carvings, carved monuments, plastered architectural facades, and masonry superstructures delineating rooms with corbel vaults. However, in southern Belize, even high-status households generally lack these types of architectural elaborations [85, 86]. Architectural elaborations do include stairways, low benches, stepped and vertical walled foundations [70], and subfloor tombs [69]. The hilly region is known for elaborate modification of hillslopes with cut-stone facings, or facades, to give the appearance of even greater investment the built environment. That level of investment is restricted to core architecture and wealthy district seats [87]. We were unable to assess variation in construction materials or the presence/absence of specific architectural elaborations due to the heavy vegetation encountered during the pedestrian survey.

In other regions of the Maya Lowlands (e.g., Copan [14]) and beyond, construction materials and architectural elaboration are often indicators of wealth differences (e.g., Hohokam, [44, 48]). In southern Belize, house size, and specifically plazuela volume, provide the best measure of wealth inequality, where the calculation of plazuela volume directly reflects the extent of the labor inputs necessary to construct platforms and modify hilltops.

#### Calculating plazuela area and volume

Gini coefficients were calculated based on three different residential dimensions: basal area (m^2^), surface area (m^2^), and volume (m^3^) for each plazuela. Basal area was calculated using the Calculate Geometry tool in ArcMap 10.7. Surface area and volume were calculated using a 1 m lidar-derived digital elevation model (DEM) based on methods adapted from Ebert and colleagues [88] and Smith and colleagues [89]. The analytical steps include:

1. Create 1 km^2^ polygons that cover the extent of settlement system
2. Clip a 1 m DEM to the 1 km^2^ polygon
3. Create a Triangular Irregular Network (TIN) from each 1 km^2^ DEM
4. Create a 3D polygon of each plazuela from the TIN
5. Add elevation information to the 3D polygons
6. Calculate the polygon volume and surface area

For comparison across Mesoamerica, volume metrics are not always available, or houses may have been built on ground surface rather than elevated platforms (e.g., Oaxaca). In those cases, we discuss and compare Gini coefficients for area (rather than volume). Although Gini values for area and volume do vary, the Gini coefficients generally indicate similar measures of wealth inequality.

**Fig 3:**
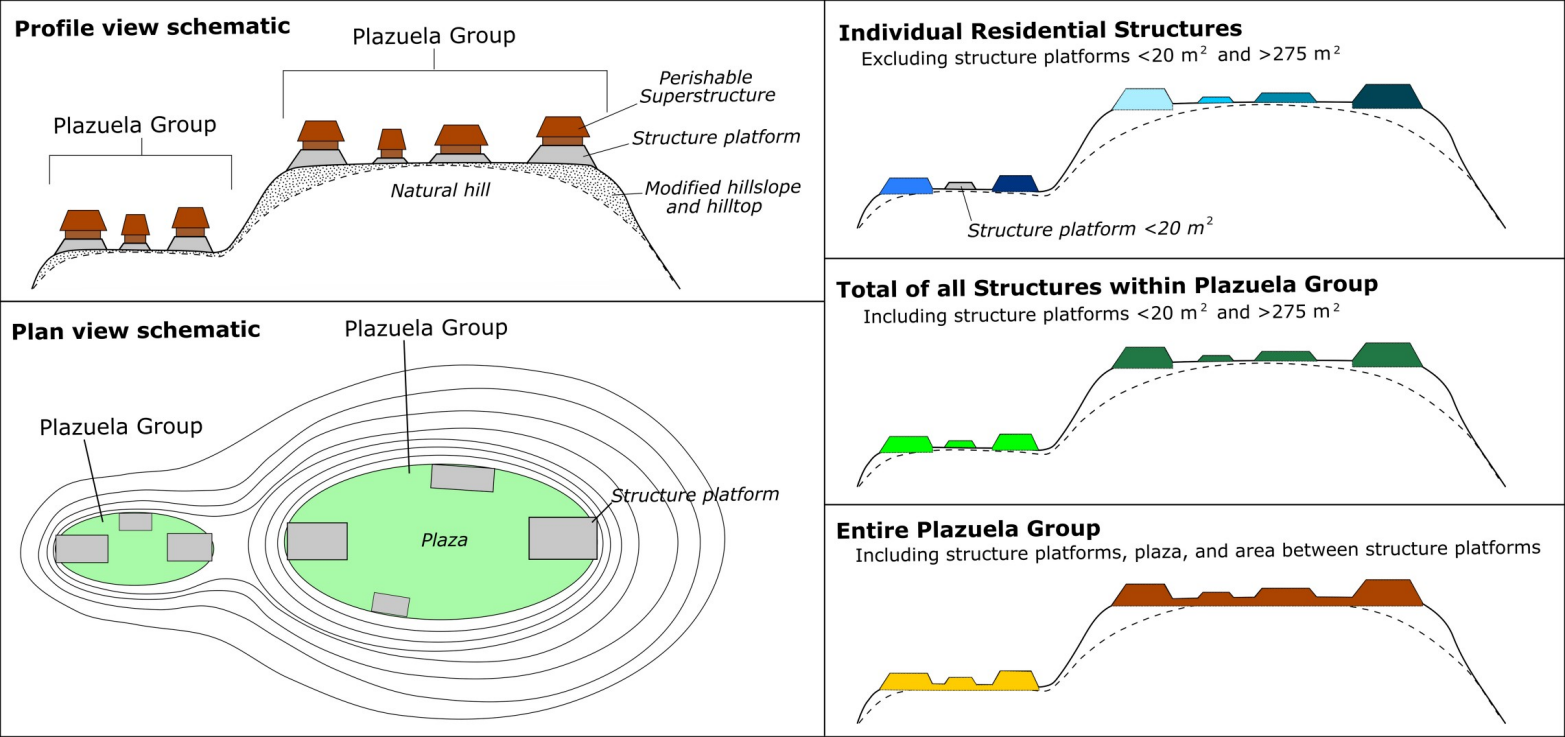
The plazuela and house size. A schematic of the plazuela as the residential unit. We use the plazuela (bottom right) as it represents the entire domestic activity area and hilltop modifications.

The Gini for the entire plazuela, which is representative of a domestic unit and includes the labor required to modify hilltops for residential construction [69, 84], are reported below. In our evaluation of wealth inequality in the Maya region, we only considered published Gini coefficients that used the entire household (plazuela or houselot) as the unit of analysis, rather than individual structures (Fig 3). Thus, we excluded published Gini coefficients reported for Sayil [34], Dzibilchaltun [37], and Komchen [43], which use the area and volume of individual structures to calculate a Gini coefficient. We do include published data that used all structures within the plazuela in regions with shallow bedrock (i.e., the Yucatan [90]) and where the measured unit contained all domestic functions (e.g., terraces in Oaxaca [3]) and multi-room household structures in Central Mexico [39]). This allows for comparable units of analysis for macro-regional comparisons of wealth inequality.

### Scales of analysis: Micro-region, polities, districts, neighborhoods

We assess wealth inequality at four nested spatial scales: (a) the micro-region of the Rio Blanco valley, (b) the polity, (c) the district, and (d) the neighborhood. The micro-region consists of all houses at Uxbenká and Ix Kuku’il. The polity encompasses houses within the boundaries of each polity (Table 1). Polity boundaries were previously determined by combining data from the pedestrian survey and conducting a k-means cluster analysis of all surveyed plazuelas (n = 302) [78]. Districts and neighborhoods were defined based on geospatial cluster and landscape analyses [see 73].

**Table 1:** Descriptive data for Uxbenká and Ix Kuku’il.

Variable	Uxbenká	Ix Kuku’il
Structures (total in plazuelas)	476	321
Settlement Groups	132	99
Household Plazuelas	180	122 / 93*
Neighborhoods	20	16
Districts	3	5
Chronology	250–900 CE	400–1000 CE
Periods	Early and Late Classic	Early and Late Classic

*At Ix Kuku’il, 29 plazuelas are north of the lidar zone and therefore surface area and volume were calculated for the lower value presented in the table.

### R: Bootstrapping the Gini coefficient

To calculate the Gini coefficient, we used the packages *reldist* [91] to calculate the Gini coefficient and *boot* [92] for bootstrapped error ranges [93 S1 Text] to accommodate small sample sizes [41]. The confidence intervals are presented as the lower and upper boundaries of the Gini coefficient. For replicability, we resampled each dataset 1000 times [94 p136] using a random number seed of 42 [following 95].

### Results: Gini coefficients at Uxbenká and Ix Kuku’il

High degrees of wealth inequality are present in all nested scales at Uxbenká and Ix Kuku’il (Table 2; for neighborhood Gini coefficients, see S2 Table). The Gini coefficients for the micro-region, the Rio Blanco valley, and for each polity are relatively high and comparable to Gini coefficients calculated for other Classic Maya centers. Variability is present between districts at Uxbenká and Ix Kuku’il and is higher for neighborhoods, which often have smaller sample sizes. Comparing the districts within each polity, those with more plazuelas settled earlier have higher Gini coefficients.

**Table 2:** Gini coefficient results.

Provenience		Gini Coefficient for Plazuela Volume (m^3^)	Bootstrapped 95% Confidence Interval for Plazuela Volume (m^3^)		Sample Size	Number of Plazuelas Occupied During Time Period^a^				
Polity Name	Nested Scale		Gini Lower Boundary	Gini Upper Boundary		lf (250 BCE—250 CE)	ec I (250–400 CE)	ec II (400–600 CE)	lc (600–830 CE)	tc (830–1000 CE)
Rio Blanco Valley	Micro-region	0.555	0.526	0.582	*273*	6	34	46	99	5
Uxbenká	Polity	0.537	0.511	0.558	*180*	5	29	37	64	3
Ix Kuku’il	Polity	0.585	0.517	0.631	*93*	1	5	9	35	2
Uxbenká	District 1	0.585	0.503	0.640	*14*	2	5	6	9	
Uxbenká	District 2	0.496	0.451	0.534	*41*	2	6	9	15	2
Uxbenká	District 3	0.410	0.363	0.454	*35*	1	9	10	16	1
*Uxbenka*	*District Mean*	*0*.*497*	na	na	*na*	* *	* *	* *	* *	* *
Uxbenká	Outside of a district	0.574	0.533	0.604	*90*		9	12	24	
Ix Kuku’il	District 1	0.680	0.570	0.740	*22*		2	3	12	1
Ix Kuku’il	District 2	0.588	0.489	0.649	*16*		2	3	5	
Ix Kuku’il	District 3	0.346	0.242	0.419	*11*			1	5	
Ix Kuku’il	District 4	0.421	0.343	0.473	*9*				2	
Ix Kuku’il	District 5	0.587	0.504	0.644	*12*	1	1	1	3	1
*Ix Kuku’il*	*District Mean*	*0*.*524*	na	na	*na*	* *	* *	* *		
Ix Kuku’il	Outside of a district	0.448	0.387	0.486	*23*			1	8	

Gini Coefficients for plazuela volume at Uxbenká and Ix Kuku’il polity and districts. lf = Late Formative; ec I = Early Classic I; ec 2 = Early Classic 2; lc = Late Classic; tc = Terminal Classic. See S2 Table for all area Gini coefficients and neighborhood volume Gini coefficients.

^a^Many plazuelas do not have temporal data

### Comparing wealth inequality across Mesoamerican polities

We found that the quantitative assessments of wealth inequality in this peripheral region are comparable to those calculated for larger Classic Maya polities located in the central heartland of the Maya world, such as Tikal [38], Caracol [35], and Uxul [42]. Uxbenká and Ix Kuku’il, while at the periphery of the lowlands, exhibit high degrees of wealth inequality. In the Rio Blanco micro-region the total Gini coefficient of all 273 plazuelas is 0.56. At the polity level, Uxbenká has a Gini of 0.54 and Ix Kuku’il has a Gini of 0.59.

The degree of wealth inequality at Uxbenká and Ix Kuku’il mirrors that of other Classic Maya polities. Caracol, a precocious polity deeply entrenched in the hypercompetitive geopolitics of the Classic Maya, has a Gini of 0.60 [35]. Other large and more densely populated Classic Maya polities have similar Gini coefficients (Palenque: 0.44 [37]; Chunchucmil: 0.56). Uxbenká and Ix Kuku’il, with an average of 23 structures/km^2^ and 15 structures/km^2^, respectively [66 Table 1.4], had high degrees of wealth inequality, comparable to polities with more densely populated settlement systems (e.g., Caracol Cohoun Ridge: 87 structures/km^2^; Chunchucmil: 350–950 structures/km^2^; and Palenque: 673 structures/km^2^ [96 Table 5.2]; Uxul: 296 structures/km^2^ [97]; Tikal core: 275 structures/km^2^ [37 p33], Tikal periphery: 145 structures/km^2^ [98]). Although varied in their settlement density but similar in their degree of wealth inequality, these polities follow the same basic sequence of growth with small populations prior to the Early Classic period and rapid population growth after 400 CE. Thus, for this sample from the Maya region, settlement density alone does not directly correlate with wealth inequality (Table 3).

**Table 3:** Comparison of Gini coefficient and settlement density.

Polity	Gini	Settlement Density (Strs/km^2^)
Uxbenká	0.54	23
Chunchucmil	0.56	350–950
Ix Kuku’il	0.59	15
Caracol	0.60	87
Uxul	0.62	296
Palenque	0.44*	673
Tikal	0.62*	145–275

Classic Maya Settlement Density and Volume Gini (*Area Gini)

Maya polities have low-density settlement systems that conform to similar patterning associated with more autocratic forms of governance [77]. Neighborhoods and districts composed of households of varying size are documented across the Maya region including Chunchucmil [30] to Caracol [99] to Copan [100]. Even densely settled Maya cities, like Chunchucmil, have more dispersed settlements than most of their northern Mesoamerican counterparts, i.e. Teotihuacan and Tenochtitlan [37].

**Fig 4:**
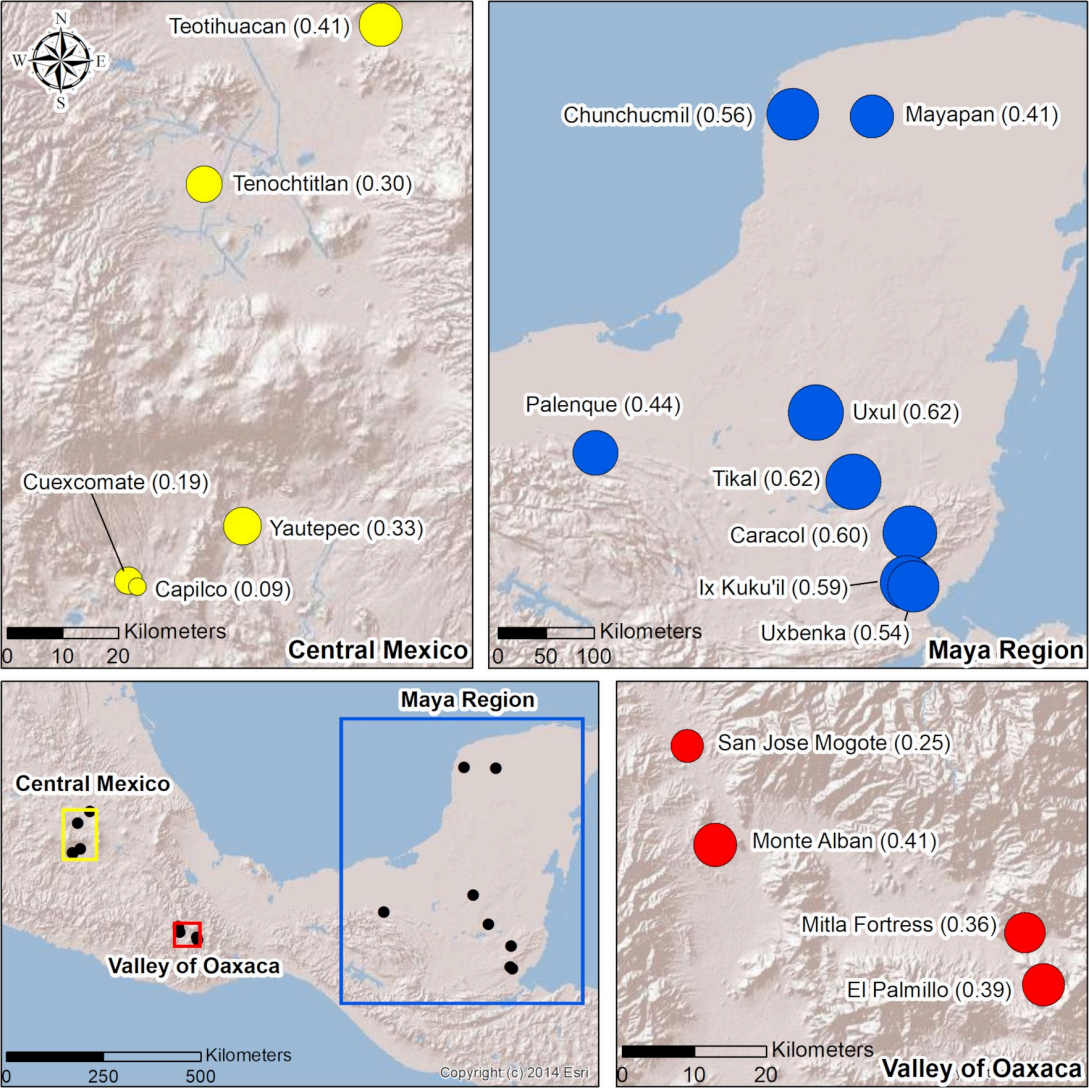
Gini coefficients in Mesoamerica. Map of Mesoamerica (lower left) displaying Gini coefficients for each community within each region (see Table 5). All markers are equally scaled with larger markers representing higher Gini coefficients (i.e., greater wealth inequality) and smaller markers indicating lower Gini coefficients. Base map images are the intellectual property of Esri and are used herein under license. Copyright 2014 Esri and its licensors. All rights reserved.

**Fig 5:**
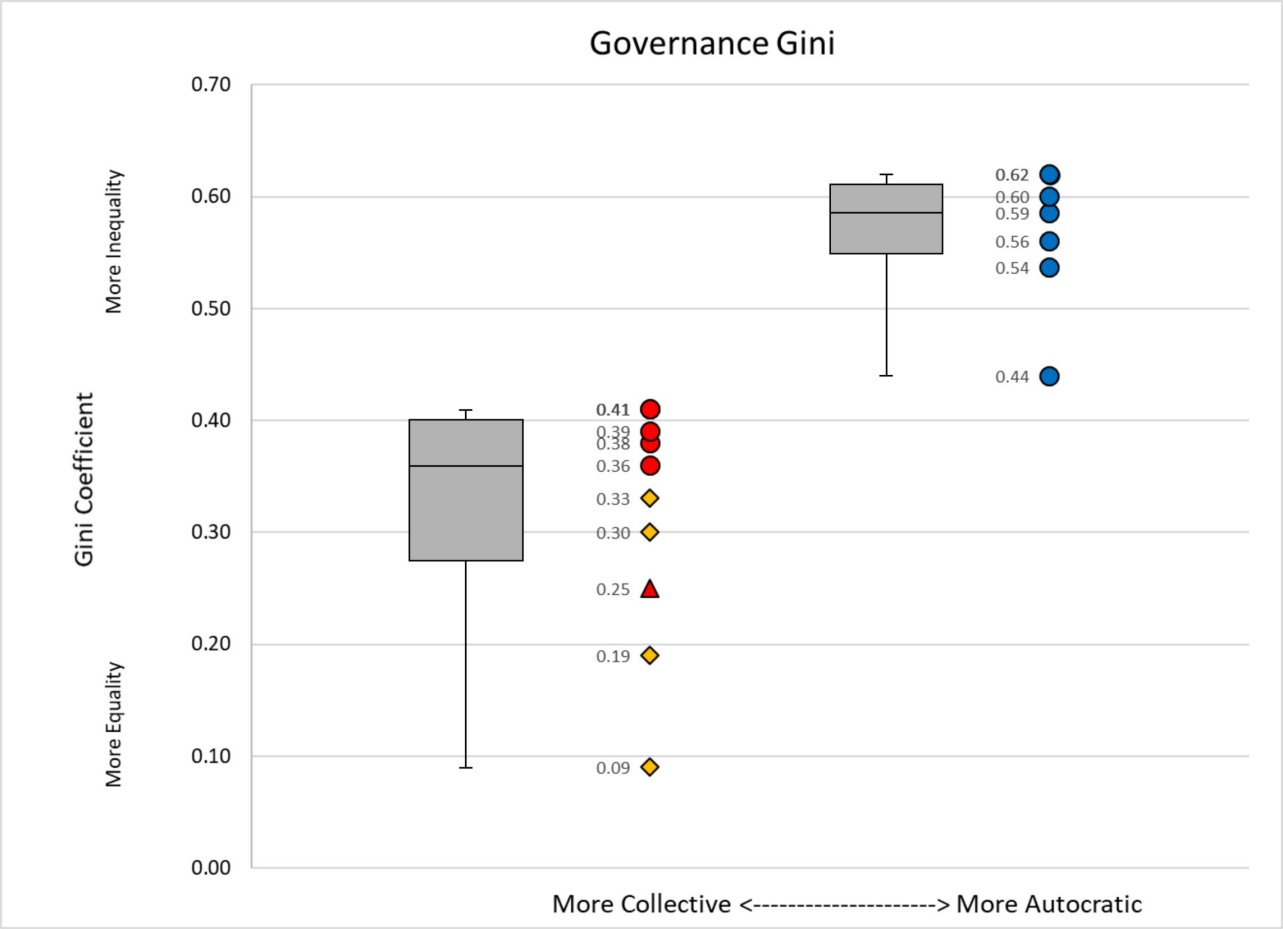
Range and variability of wealth inequality between more collective and more autocratic states. Box and whisker plots with the median and error bars are on the left and data points are displayed on the right. Region is designated by color: Maya (Blue), Oaxaca (Red), and Central Mexico (Yellow). Time period is designated by shape: Formative (Triangle), Classic (Circle), and Postclassic (Diamond).

Maya polities generally exhibit higher degrees of wealth inequality than contemporaneous polities in Central Mexico or Oaxaca (Table 4; Figs 4 and 5). Maya polities cluster together, with Gini coefficients ranging from 0.44–0.63 (Table 5) with an average Gini of 0.55 (Table 4). Other Mesoamerican polities exhibit greater variability and generally lower Gini coefficients. Specifically, communities in Oaxaca have a tighter range (0.25–0.41) and higher average Gini (0.36) than polities in Central Mexico (range: 0.09–0.41; mean: 0.26).

**Table 4:** Mesoamerican polity Gini coefficients.

Variable		Gini Mean	Gini Median	*Sample Size*
Region	Maya	0.55	0.57	*8*
	Central Mexico	0.26	0.30	*5*
	Oaxaca	0.36	0.38	*5*
Political Organization	More Autocratic	0.57	0.59	*7*
	More Collective	0.32	0.36	*11*

Mean and median Gini coefficients for regions and political organization in Mesoamerica.

**Table 5:** Gini coefficients for area and volume at Mesoamerican communities.

Provenience Information						Gini Coefficient		Additional Information	
Polity	Region	Political Organization	Dates	Period	*Sample size*	Area	Volume	Notes	Source
Capilco	Central Mexico	More collective	1480 CE	Late Postclassic B	*21*	0.16	0.09	Individual households (multi-room structures)	[4] Tables 11.1 and 11.3, [39]
Cuexcomate	Central Mexico	More collective	1480 CE	Late Postclassic B	*135*	0.25	0.19	Individual households (multi-room structures)	[4] Tables 11.1 and 11.3, [39]
Yautepec	Central Mexico	More collective	1480 CE	Late Postclassic B	*1*,*619*	0.21	0.33	Individual households (multi-room structures)	[4] Tables 11.1 and 11.3, [39]
Uxbenká	Maya: Southern Belize Zone	More Autocratic	250–800 CE	Classic	*180*	0.38	0.54	Surface area and volume of household plazuelas	This study
Chunchucmil	Maya: Northern Plains	More Autocratic	400–800 CE	Early Classic and Late Classic	*411*	0.34	0.56	Total area and volume of architecture in EC houselot and LC platforms + structures	[90] Appendix 5.3, this study using [101] Appendix 1
Ix Kuku’il	Maya: Southern Belize Zone	More Autocratic	400–800 CE	Classic	*94*	0.40	0.59	Surface area and volume of household plazuelas	This study
Caracol	Maya: Central Zone	More Autocratic	600–900 CE	Late Classic	*4*,*058*	0.34	0.60	Plazuela (household) area and volume	[35]
Uxul	Maya: Campeche	More Autocratic	600–800 CE	Late Classic	*243*		0.62	Construction volume by patio groups	[42]
San Jose Mogote	Oaxaca	More collective	1100–700 BCE	Formative	*1*,*000*	0.25		-	[4]
Tenochtitlan	Central Mexico	More collective	1500 CE	Contact	*30*,*006*	0.30		-	[4] Tables 11.1 and 11.3
Mitla Fortress	Oaxaca	More collective	500–850 CE	Classic	*517*	0.36		Terrace areas (similar to plazuela or house lot)	[3]
Valley of Oaxaca sites	Oaxaca	More collective	500–850 CE	Late Classic	*39*	0.38		Patio area	[3, 4] Table 11.1
El Palmillo	Oaxaca	More collective	500–850 CE	Classic	*1*,*343*	0.39		Terrace areas (similar to plazuela or house lot)	[3]
Mayapan	Maya: Northern Plains	More collective	1200–1450 CE	Late Postclassic	*4*,*031*	0.41		Household Area	[4] Tables 11.1 and 11.3
Monte Alban	Oaxaca	More collective	500–850 CE	Classic	*22*	0.41		Patio area	[3]
Teotihuacan	Central Mexico	More collective	400–500 CE	Early Classic	*15*,*495*	0.41		Individual households (multiroom apartments within larger compound)	Michael E. Smith, personal communication
Palenque	Maya: Northwest Zone	More Autocratic	600–800 CE	Late Classic	*1*,*135*	0.44		House area	[34, 37] Table 5.1
Tikal	Maya: Central Zone	More Autocratic	765 CE	Late Classic	*756*	0.62		Household Area	[4] Tables 11.1 and 11.3

Gini coefficients for political centers across Mesoamerica.

We define “more autocratic” (less collective) and “more collective” based on published studies [9, 28]. Classic Maya polities were, generally, autocratic compared to most of the other Mesoamerican centers [9]. Relative degrees of wealth inequality correlate with modes of governance at other premodern polities [8, 102]. In a cross-cultural sample drawn from cases in the old and new world, the degree of wealth inequality correlated with political organization, so that more autocratic polities had higher Gini coefficients than those that were more collectively organized [4, 102]. For Mesoamerican polities, those led by autocratic regimes, where power and wealth were concentrated, seem to have greater wealth disparities than more collectively organized polities [9].

External trade was fundamental for financing Maya rulers [103] who highly valued their participation in the prestige economy [104, 105] and where networked principals [106] used high-status items to symbolize their power and authority. From the top-down, the monopolization and control of exchange routes and networks were fundamental to power relations [9, 14, 107]. In contrast, the more collectively governed polities in our sample (Table 4; Figs 4 and 5) were more reliant on internal resources, the local production of agrarian and craft goods [108], and, in general, lower Gini values were recorded for these polities [102]. To further examine the link between the exclusionary, top-down control of external trade networks and degrees of wealth inequality, we assess patterns of wealth inequality in southern Belize at four nested scales.

### Intra-settlement wealth inequality at Uxbenká and Ix Kuku’il

Across Mesoamerica we observe a relationship between modes of governance and degrees of wealth inequality [4]. Specifically, polities in which principals relied on the external resources, such as the monopolization of trade networks, were characterized by higher degrees of inequality than polities in which fiscal financing was based on internal resources. In the Maya region, highly crafted prestige goods and valued raw materials were distributed through exchange networks [109–111] that linked the powerful polities in the western Maya area, including the Central Petén [10] and along the Usumacinta River [103, 107, 112]. Although glyphic texts provide few direct indications that Maya polities in the east participated in these principal alliance and exchange networks [113], evidence for long distance political connections nonetheless exists for some eastern Maya polities including those in southern Belize [56, 59, 106].

Below, we focus on wealth inequality in the Rio Blanco valley micro-region of southern Belize. Can we gauge how access to networks through which goods were transferred affected wealth inequality at the peripheral polities of Uxbenká and Ix Kuku’il? Is differential access to exchange networks at Uxbenká and Ix Kuku’il reflected in intra-community wealth inequality? To assess this question, we consider three key variables: (a) proximity to a known trade route that passes through Uxbenká, (b) the relative priority of occupation at different sectors (i.e., districts and neighborhoods) of these polities, and (c) architectural and artifactual evidences of organizational variability and patterns of access among the multi-nested units. For example, were sectors of these centers associated with stelae and elite funerary contexts characterized by higher degrees of inequality?

**Fig 6:**
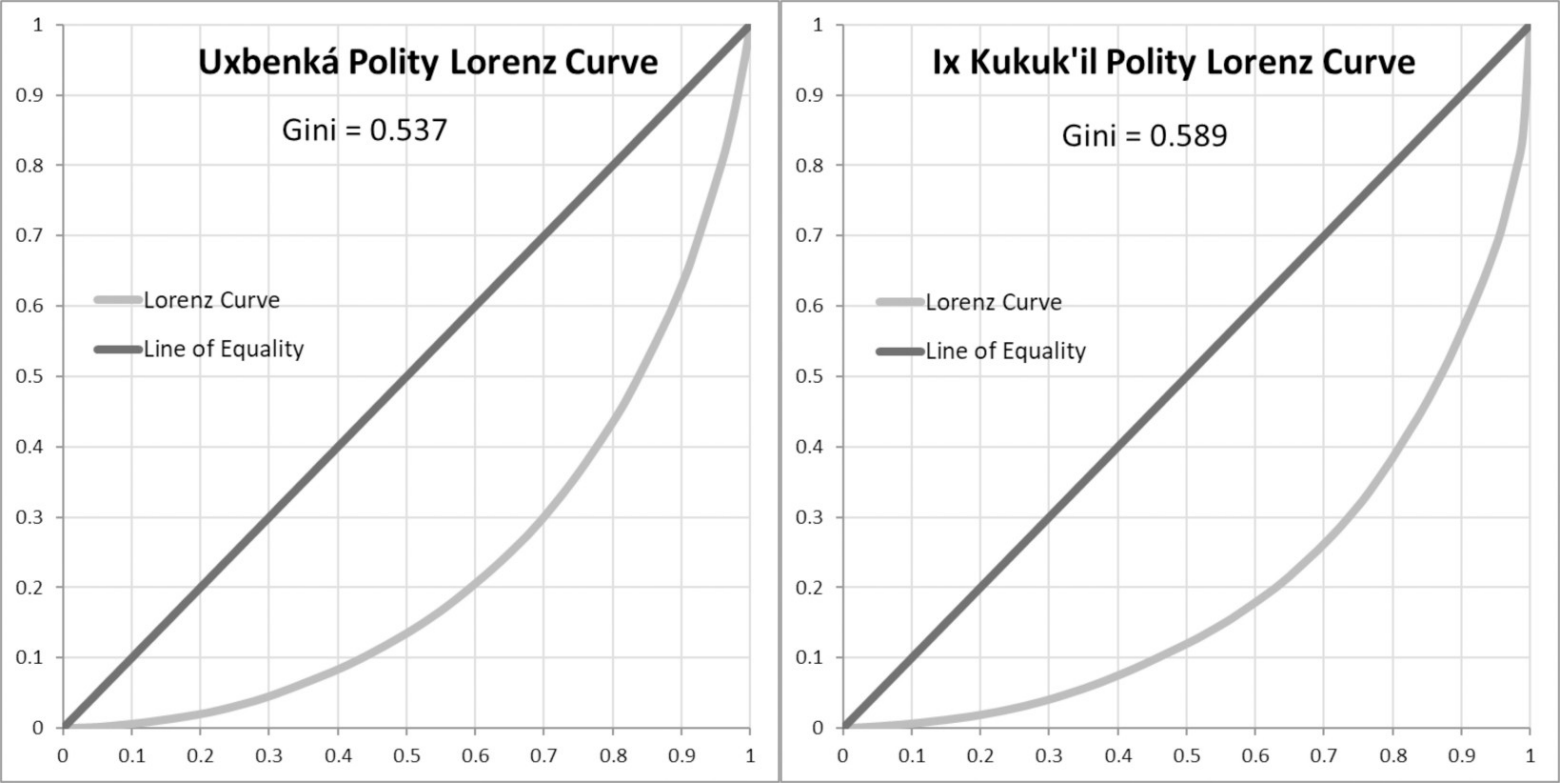
Lorenz curves. Lorenz curves showing similar distributions of house size for Uxbenká and Ix Kuku’il. (Lorenz curves created with assistance from A.S.Z. Chase).

Despite their peripheral positioning in the Maya world, relatively high degrees of wealth inequality were present at Uxbenká and Ix Kuku’il (Table 2; Fig 6). Higher degrees of wealth inequality were present in districts that had strong connections to external trade. For example, at Uxbenká, District 1 may have generated wealth through its connections with the Petén as early households mediated trade during the founding of the district. Hieroglyphic texts on Stela 11 (in District 1) mention the 14th ruler of Tikal, *Chak Tok Ich’aak* I (Jaguar Paw I), and affirm a relationship between the incipient principals of Tikal and Uxbenká [59] during the Early Classic, when Tikal was heavily vested in a north-south trade route to Copan [114]. This connection with Tikal and the greater Petén region may have solidified trade during the Late Formative rise of Uxbenká [87], allowing principals to control the trade route and facilitate the movement of goods from east to west. Over centuries, principals brokered and garnered that material wealth into greater degrees of wealth inequality [72]. In each district, and to some degree in the neighborhoods, smaller households are clustered around a larger household, supporting vertical patron-client type relationships.

**Fig 7:**
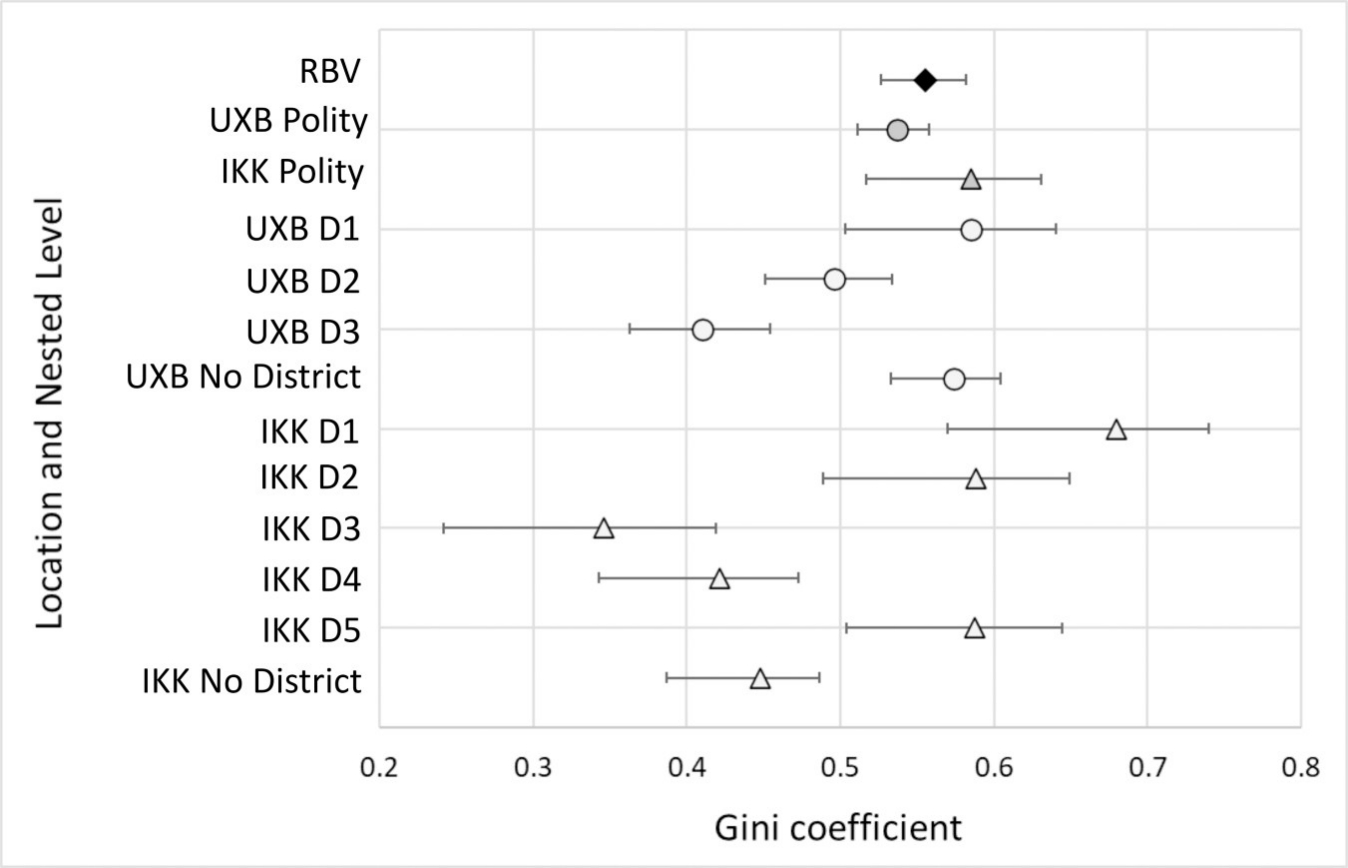
Multi-scalar house size Gini coefficients. Gini coefficients (shapes) with bootstrapped 95% confidence intervals (whisker lines) for the micro-region of the Rio Blanco valley (RBV, black diamond), polity (grey), and district (white) of Uxbenká (UXB, circle) and Ix Kuku’il (IKK, triangle). The micro-region and polities have higher wealth inequality and the Gini coefficients in districts are more varied.

**Fig 8:**
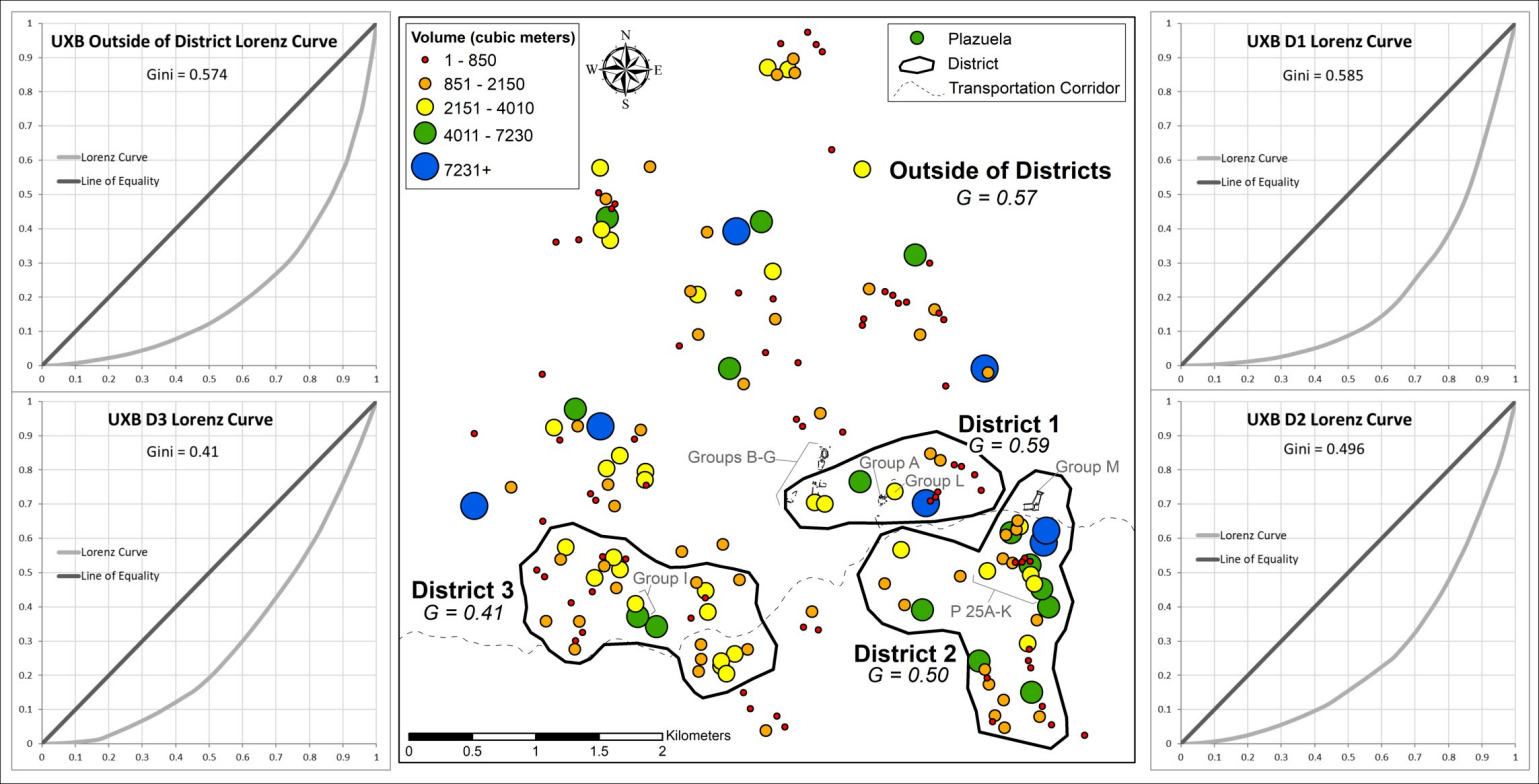
Uxbenká district level Gini coefficients. Circles represent the volume of plazuelas (based on natural breaks classification). Groups and plazuelas (P) mentioned in the text are labelled. Lorenz Curves visually represent the distribution of inequality for each district and for households outside of a district. (Lorenz curves created with assistance from A.S.Z. Chase).

**Fig 9:**
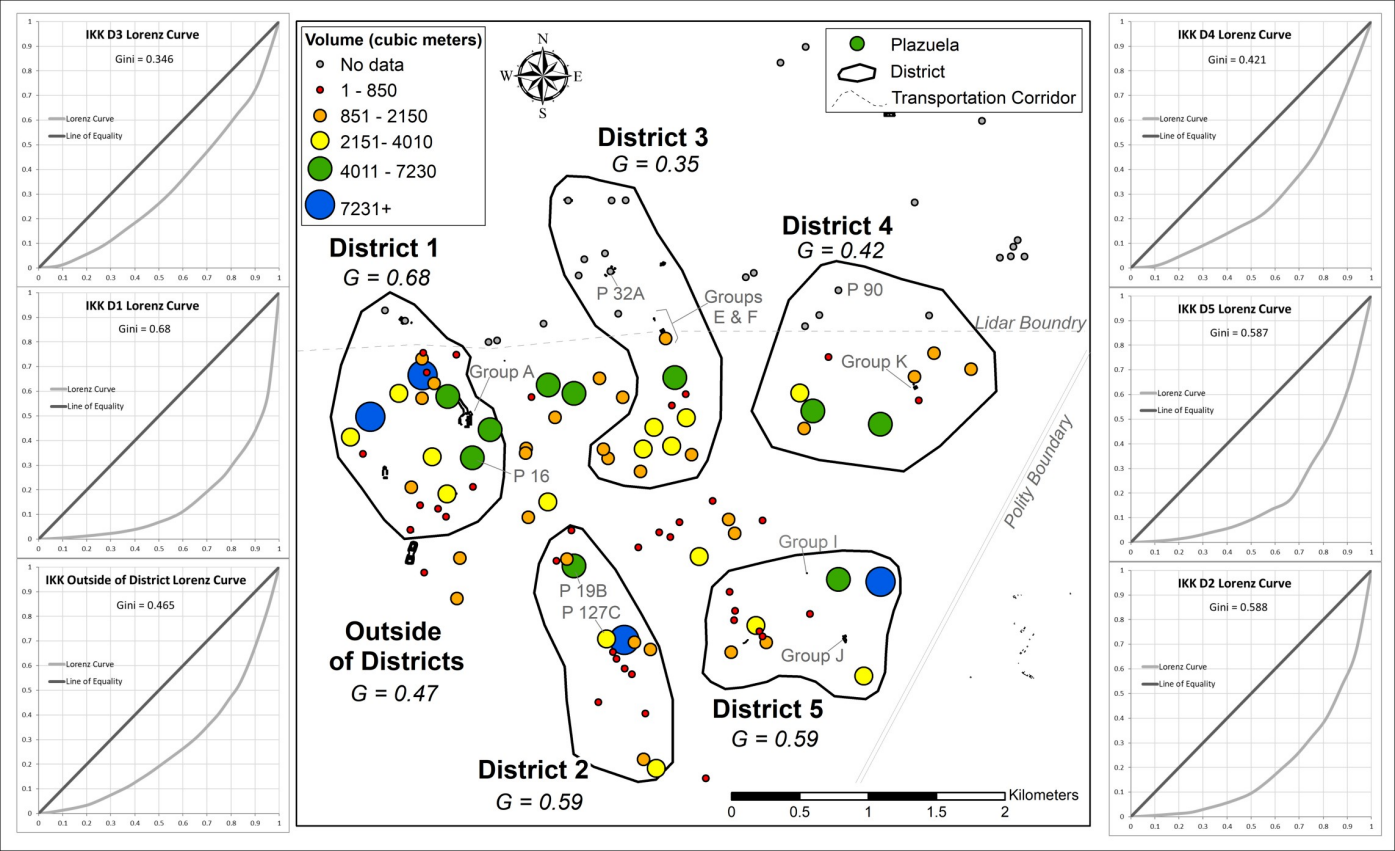
Ix Kuku’il district level Gini coefficients. Circle size represents the volume of plazuelas (based on the Uxbenká natural breaks classification for ease of comparison). Groups and plazuelas mentioned in the text are labelled. Lorenz Curves visually represent the distribution of inequality for each district and for households outside of a district. (Lorenz curves created with assistance from A.S.Z. Chase).

Within districts, high degrees of wealth inequality were present at both Uxbenká and Ix Kuku’il. Districts with higher Gini coefficients were occupied earlier and situated more proximate to the trade route. At Uxbenká, the degree of wealth inequality was highest at District 1 (0.59), followed by District 2 (0.50), then District 3 (0.41) (Table 2; Figs 7 and 8). All three districts were founded by 300 CE and are situated along the trade route. Imported prestige goods (e.g., jade, conch shells, polychrome pottery) are present in elaborate, elite funerary contexts in all three districts [81]. Comparatively, wealth inequality was more variable at Ix Kuku’il, with Gini coefficients ranging from 0.35 (District 3) to 0.68 (District 1). In addition to District 1, Districts 2 and 5 had high degrees of wealth inequality (District 2, 0.59; District 5, 0.59) while District 4’s Gini coefficient was lower (0.42; Fig 9). At Ix Kuku’il, the foundation of districts occurred over several centuries. But districts with higher Gini coefficients had larger populations earlier in the polity’s history than those with lower Gini coefficients (Table 2). Districts with high wealth inequality are closer to Uxbenká and are situated along the Ix Kuku’il-Uxbenká least-cost path, representing possible trade networks.

**Fig 10:**
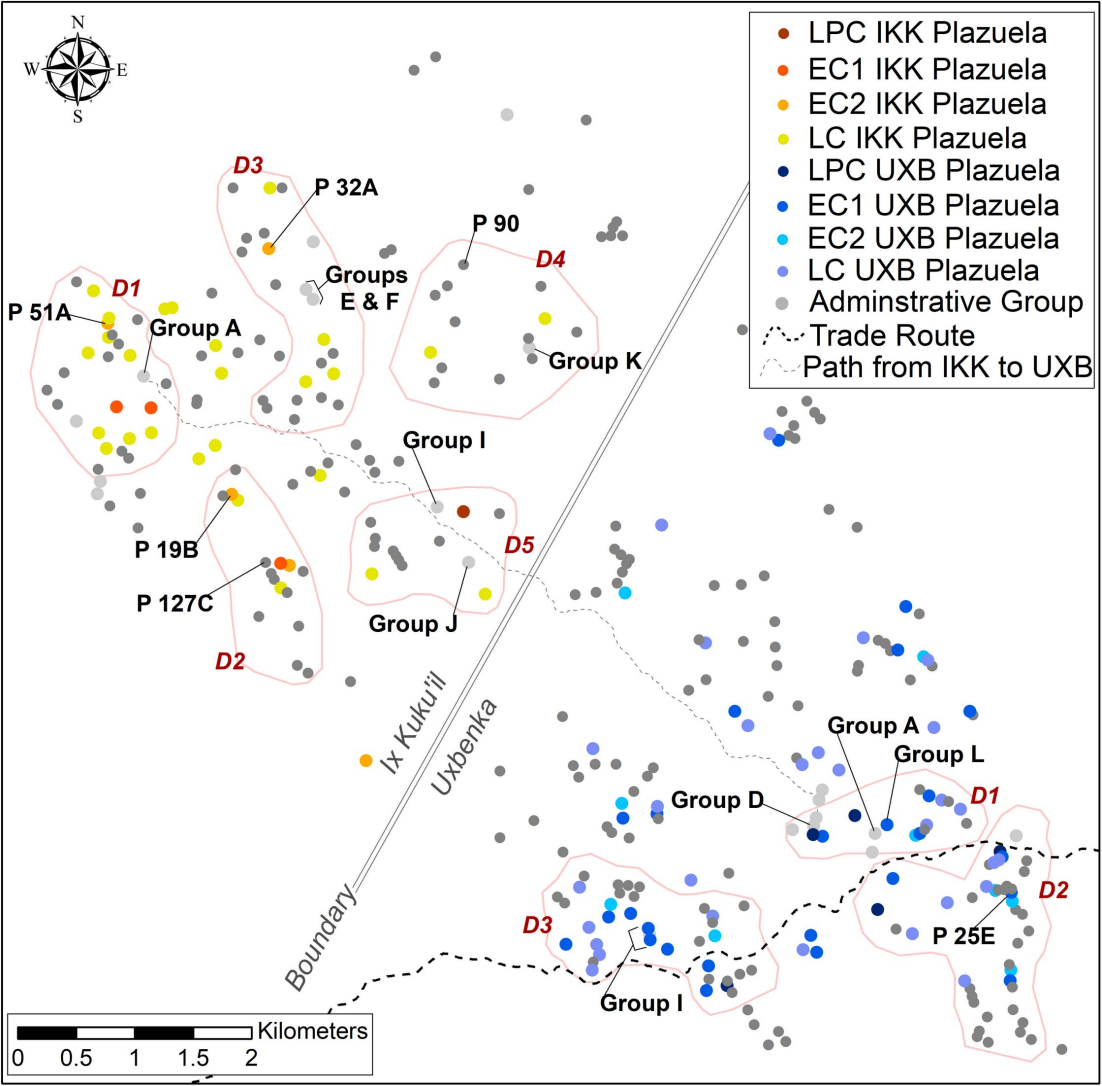
Occupational priority at Uxbenká and Ix Kuku’il. Foundation date of plazuelas are indicated by the corresponding color. Plazuelas and groups mentioned in the text are highlighted. Districts are outlines in pink.

Occupational priority is linked to higher degrees of wealth inequality across the districts of the Rio Blanco valley. However, at the polity scale occupational priority did *not* affect wealth inequality. Uxbenká had a substantially larger population centuries earlier than Ix Kuku’il ([78], Fig 10), yet their Gini coefficients are similar (Figs 6 and 7). At Uxbenká and Ix Kuku’il, district level Gini coefficients correlate positively with early population expansion (Table 2). At Ix Kuku’il, districts with houses founded before 400 CE (Districts 1, 2, and 5) exhibit greater wealth inequality than districts founded after 400 CE (Districts 3 and 4; Table 2). The older households are near the least-cost path between Uxbenká and Ix Kuku’il, which would have provided early occupants with access to trade goods.

Greater architectural investments and prestige goods are more abundant in districts with higher Gini coefficients. At Uxbenká, wealth inequality is reflected in massive landscape modifications in all three districts [84] and elaborate tombs are located in the district seats. These larger, high-status households had access to luxury goods from the Guatemalan highlands, Belize River Valley, the Petén, and coastal regions [115] including carved jade, modified conch shells, and polychrome pottery, which are less abundant in non-elite houses. District 1 has the highest Gini coefficient and the most architecture associated with power and authority. The royal tomb, located in District 1, has a staired entryway and was reentered throughout the 6^th^ and 7^th^ centuries CE. The abundance of polychromes in District Seats is significantly higher than polychromes in smaller houses. Other high status groups have imported polychrome vessels, figurines, conch shells, eccentric flint, bone lip plugs, shell beads, and jade earspools (Table 6) and incorporate iconography associated with wealth and power [81] attesting to the accumulation of goods through patron-client relationships.

**Table 6:** Imported and luxury artifact data for Uxbenká.

District	District Gini	Neighborhood	Neighborhood Gini	SG	Plazuela	Jade / Greenstone Count^a^	Marine Shell Count	Obsidian Count	Adornments Description (counts for jade and marine shell included in columns to the left)
1	0.585	6	0.538	37	a			16	
				37	b	1		3	
				37	d	1		5	jade earspool, 1 stone bead
				L		7	9	159	4 jade beads, 3 shell beads, 1 bone lip plug
		7	0.107	21				41	1 ceramic bead
				F		2	1	65	
		8	0.459	22				6	
				26	a			27	
				26	b			15	
				27				1	
2	0.496	4	0.301	5			1	8	
				13				27	
				18				24	
				29				7	
				63				1	
		5	0.51	10		1		29	1 jade earspool, 1 ceramic earflare
				25	b			1	
				25	d			21	
				25	e	4	24	101	1 jade bead, 1 jade pendant, 1 jade earspool, 2 shell beads, 1 starburst bone pendant, 1 bone lip plug, 1 bone bead, 1 stone bead, 1 unspecified bead
				25	g	1	2	9	2 shell beads
				25	h			4	1 ceramic bead
				28	a	1	1	5	
				28	b			26	
		14	0.454	47				1	
				81 / 2				3	
3	0.41	2	0.45	3			2	8	1 shell bead
				50			1		1 shell pendant
				51				4	
				52				10	
				53				12	
				54				22	
				60				23	
				I	b	7	6	22	3 jade beads, 2 jade earflares, 4 shell beads
		3	0.343	4				45	
				62				39	1 bead, 1 ceramic earflare
				79	b			1	
				79	d			16	
-	0.574	1	0.459	1				2	
				87			1	2	
		9	0.521	23				2	
				24				7	
		10	0.34	30				11	
				84		1		2	
		11	0.55	33			1		
				42				14	1 figurine bead
				43				4	
				44				4	
		12	0.574	35				1	
				45				46	
		13	0.358	36		1		24	2 stone beads, 1 black stone pendant, 1 greenstone bead
				38				69	
				39				24	
		15	0.493	89				11	
		-		X134				1	
		-	-	116				1	

Tabular artifact data for Uxbenká divided by districts. Greater differences in access to resources are present in districts with higher Gini coefficients. Many households had access to luxury and imported goods through a range of vertical and horizontal networks.

^a^Jade counts may be underrepresented due to looting activity of tombs, particularly in L, 25, and I.

Uxbenká Districts 2 and 3 contain hallmarks of elite power but less so than what is present in District 1. In District 2, public architecture was constructed around 400 CE and larger houses had imported goods including polychrome pottery [116], jade beads, and a carved jade pendant with *kin’ich ajaw* (sun lord / ruler) imagery [81]. In District 3, which has the lowest Gini coefficient of the Uxbenká districts, elaborate constructions are markedly smaller than those noted in District 1 [89 Appendix 2]. Prestige goods from an elite tomb include imported polychrome pottery [115] and personal adornments including jade earspools, a zoomorphic jade bead, and shell beads (see Table 6).

In general, elite civic ceremonial architecture is notably smaller at Ix Kuku’il than Uxbenká, possibly because Ix Kuku’il’s nobles lacked the early political clout to undertake massive anthropogenic landscape modifications and constructions like those seen at Uxbenká. Nonetheless, the residents of Ix Kuku’il lived in houses of varying size and had differential access to prestige goods. District 1 has the highest Gini coefficient based on house size and the most monumental architecture at Ix Kuku’il [89 Appendix 2]. The principals of District 1 likely maintained a monopoly on access to imported goods based on disproportionate amounts of obsidian compared to other districts, the presence of a possible obsidian workshop [82], and other imported goods including jade (Table 7) and polychrome pottery.

**Table 7:** Imported and luxury artifact data for Ix Kuku’il.

District	District Gini	Neighborhood	Neighborhood Gini (*area Gini)	SG	Plazuela	Jade / Greenstone Count	Marine Shell Count	Obsidian Count	Adornments Description (counts for jade and marine shell included in columns to the left)
1	0.68	1	0.60	6				10	
				51	a	2		7	
				51	b			1	
		3	0.51	16		1		16	
2	0.59	-		19	b	1	1	16	1 earspool (marine shell)
		-		127	a		1	10	
		-		127	b			2	
3	0.35	7	0.46*	32	a			1	
5	0.59	12	0.36	59			1	2	2 small earspools or nose plugs (unk material), 1 bone bead
-	0.45	9	0.42	52				1	
		-		47				1	

Tabular artifact data for Ix Kuku’il divided by districts. Greater differences in access to resources are present in districts with higher Gini coefficients. Many households had access to imported goods through a range of vertical and horizontal networks.

More modest civic-ceremonial architecture is present in Districts 2 through 5. Although few tombs have been identified at Ix Kuku’il, those documented are in Districts 1 and 4. The presence of exotic and valuable goods also is evidenced at Ix Kuku’il. Personal adornments were found in Districts 2 and 5, which also contained small pieces of jade and a polychrome cylinder vase, respectively (Table 7). Polychrome pottery was found in Districts 3 and 4, but other luxury goods including jade, marine shells, or personal adornments have not yet been identified. We note that Ix Kuku’il districts with higher degrees of wealth inequality had larger civic ceremonial architecture (District 1) and greater access to prestige goods including obsidian, jade, personal adornments, and polychrome pottery.

**Fig 11:**
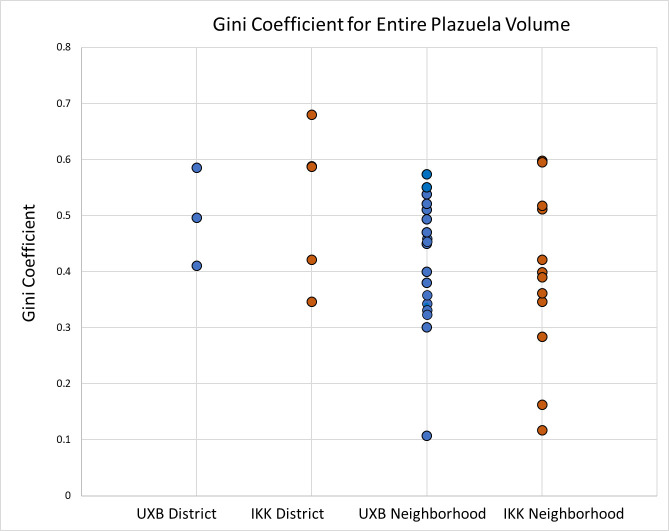
Range and variability of Gini coefficients within Uxbenká and Ix Kuku’il districts and neighborhoods. Ix Kuku’il exhibits greater variability than Uxbenká. (0 = greater equality. 1 = greater inequality).

**Fig 12:**
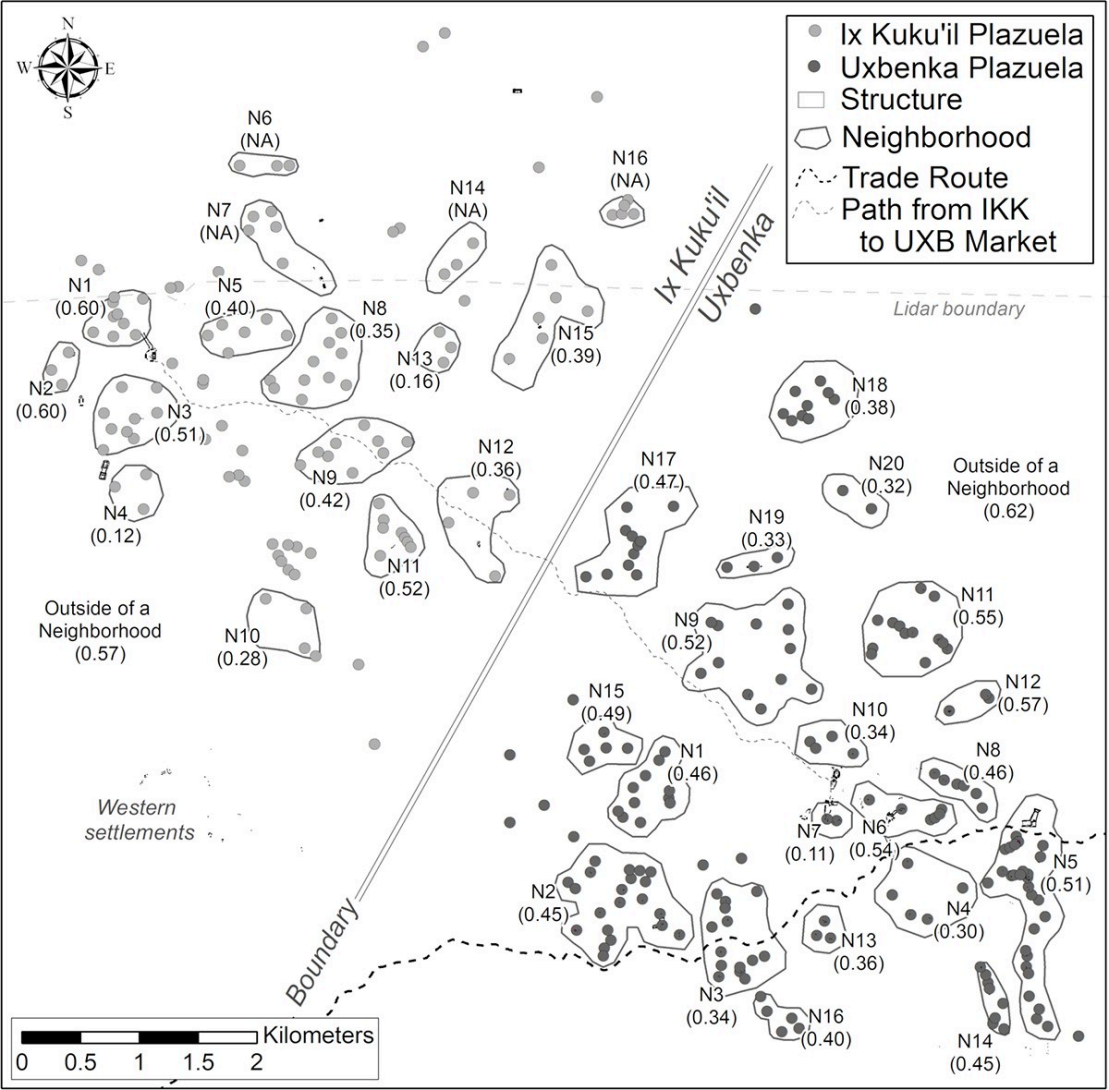
Neighborhood Gini coefficients at Uxbenká and Ix Kuku’il. Each neighborhood is labeled (e.g., Neighborhood 1 = N1) with its Gini Coefficient. Ix Kuku’il neighborhoods north of the lidar boundary do not have Gini coefficients for plazuela volume.

Wealth inequality was more variable at the district level than at the polity or micro-region level. This pattern may reflect that nobles (in district seats) lived in markedly smaller houses than rulers. Ix Kuku’il District 3 and 4 and Uxbenká District 3 have the lowest Gini coefficients and the largest houses in these districts are smaller than the largest households in other districts (Figs 8 and 9). These multi-scalar trends of wealth inequality also are present at the scale of neighborhoods (Figs 11 and 12), although more variability is likely in part due to the small sizes of the sample units.

Nobles living in district seats and lower-level neighborhood heads reaped the benefits of patron-client-like relationships and the associated trickle down of goods acquired through exclusionary exchange networks. In hinterland neighborhoods exterior to districts, larger houses had access to prestige goods. For example, at Uxbenká, households in Neighborhoods 11 (0.55) and 15 (0.49) had Pachuca obsidian. These interpersonal ties linked principals at the polity level, to nobles at the district level, and to larger, founding households at the neighborhood level [cf. 17], resulting in a trickle-down effect of wealth inequality at nested scales.

Classic Maya principals depended on transactional ties of alliance, exchange, and marriage to knit together socioeconomic networks [10]. Situated at each network node, which were linked through both horizontal (relatively equivalent) and vertical (power differentiated) ties, were Maya rulers of different rank and clout in conjunction with their followers [18]. Uxbenká and Ix Kuku’il were linked to vast inland and coastal trade networks. In addition to non-perishable prestige goods, we suspect that cloth was imported into the region, as part of these exclusionary exchange networks [117]. Historically, cloth products including mats and *huipiles* were traded to southern Belize by merchants and cacao was exported [69]. For the Classic Maya, Baron [56] points to southern Belize as an intensive cacao producing region, where networked principals hosted foreign merchants and exported cacao. Cacao served both as a ritual beverage of symbolic value and as a currency throughout Mesoamerica [118]. In southern Belize, export of cacao likely helped enrich certain households in these districts and returned valued goods from the Classic Maya heartland [56].

For the rural polities in southern Belize, patterns and degrees of wealth inequality parallel those found at core polities such as Caracol, Tikal, and Uxul in the Maya heartland. At Uxbenká and Ix Kuku’il, high degrees of wealth inequality were manifested at multiple, smaller, socio-spatial scales for each polity, although greater variability is present in the neighborhoods (Figs 11 and 12; S2 Table). Similar findings have been reported at Chunchucmil. There, Gini coefficients calculated for two of the settlement’s neighborhoods paralleled that for the polity as a whole [30]. Thus, in two peripheral domains in the Classic Maya world, northern Yucatán and southern Belize, high degrees of wealth inequality were maintained outside the Central Petén heartland. In conjunction, these case studies highlight a clear rationale to assess degrees of wealth inequality across multiple analytical scales to understand how inequities were reproduced and maintained.

## Conclusion

Inequality is ever-present and its manifestations markedly variable. To begin to understand this variation, we must build a wide, diverse set of cases, past and present. Here, we measured wealth inequality in the Rio Blanco valley at the edges of the Classic Maya world. We recorded high degrees of wealth inequality that were not entirely expected given the peripheral setting. The levels of inequality measured parallel values that have been calculated for larger contemporaneous polities situated in the Classic Maya heartland. The high degree of wealth inequality encountered is grounded in how power was financed, mainly through external resources [19]. More specifically, the monopolization of exclusionary exchange networks and the precious goods that were transported through them underpinned the wealth and power of Classic Maya principals, even at the peripheries. These political practices, and the parallel manifestations of high degrees of wealth inequality, provide a clear contrast with the levels of wealth inequality and the ways that governance and power were financed in other prehispanic Mesoamerican centers (Fig 5).

History does not repeat, but it often rhymes. In the Classic Maya world, wealth inequality emerged through the monopolization of resources and the manipulation of those resources through principal networks, which ramified those inequities at regional, polity, and neighborhood scales. Differential access to resources based on fiscal financing was a key variable in the high degrees of wealth inequality among the Classic Maya while increased collectivity and community access to public goods and services likely dampened the levels of wealth inequality among contemporaneous Central Mexico and Oaxaca communities [9, 19]. Understanding how variations in governance and financing fostered differential degrees of wealth inequality in the past elucidate how these same processes may manifest today and helps to guide policies and practices that can be implemented to lessen and prevent the further expansion of systemic inequalities in contemporary contexts.

## Supporting information

S1 Table Plazuela data. Plazuela data (area [m^2^] and volume[m^3^]) for Uxbenká and Ix Kuku’il. (See S1 Text for table metadata).
(XLSX)

S2 Table Multi-scalar Gini coefficients. Gini coefficients for the Rio Blanco Valley micro-region and the polity, district, and neighborhood scales at Uxbenká and Ix Kuku’il. Gini coefficients with 95% confidence intervals are presented for the area, surface area, and volume of plazuelas. The number of plazuelas dating to each time period links occupational priority with the Gini coefficients. (See S3 Text for table metadata).
(XLSX)

S1 Text Metadata for S1 Table. (PDF)

S2 Text R code. (PDF)

S3 Text Metadata for S2 Table. (PDF)
