# Supplementary material for: Assessing Classic Maya multi-scalar household inequality in southern Belize
Source: PLoS One. 2021 Mar 24;16(3):e0248169. doi: 10.1371/journal.pone.0248169 (PMC7990175; doi:10.1371/journal.pone.0248169)
Supplement: S2 Text — (PDF) [file pone.0248169.s004.pdf]

S2 Text: R Code (adapted from Abeles and Conway 2020: Text S1)

Uses R packages ‘reldist’ (Handcock 2016) and ‘boot’ (Ripley 2020). Set.seed determined based on Carlson (2017). Bootstrap resampling of 1000 determined based on Drennan (2009). “A” and “B” can be swapped out for different names (e.g., “A” could be “data”). Enter data into the R Console if using the RGui rather than RStudio.

Set-up:

```
install.packages("reldist")
library(reldist)
install.packages("boot")
library(boot)
```

Gini Calculation:

```
A <-c(vol1, vol2, vol3, vol4…voln)
gini(A)
set.seed(42)
B <-boot(A,gini,1000)
quantile(B$t, probs=c(0.025,0.975))
```

References

Abeles J, Conway DJ. The Gini coefficient as a useful measure of malaria inequality among populations. Malaria Journal. 2020 Dec 2;19(1):444.

Carlson DL. Quantitative Methods in Archaeology Using R. Cambridge, United Kingdom ; New York, NY: Cambridge University Press; 2017. 452 p

Drennan RD. Statistics for Archaeologists: A Common Sense Approach. 2nd ed. Springer US; 2009. (Interdisciplinary Contributions to Archaeology).

Handcock MS. Relative Distribution Methods [Internet]. 2016. Available from: http://www.stat.ucla.edu/~handcock/RelDist

Ripley B. Bootstrap Functions (Originally by Angelo Canty for S). 2020.
