# Supplementary material for: Assessing Classic Maya multi-scalar household inequality in southern Belize
Source: PLoS One. 2021 Mar 24;16(3):e0248169. doi: 10.1371/journal.pone.0248169 (PMC7990175; doi:10.1371/journal.pone.0248169)
Supplement: S3 Text — (PDF) [file pone.0248169.s005.pdf]

S3 Text: Metadata for S2 Table

Polity Name*:* Uxbenká or Ix Kuku'il, which are both in the Rio Blanco Valley.

Level*:* Nested-scale of community from largest (Micro-region) to smallest (Neighborhood).

Plazuela Area (m^2): Gini coefficient for the basal area data based on plazuelas within each nested level.

Lower Boundary: Lower Boundary of Gini based on bootstrapped error ranges with a 95% confidence interval.

Upper Boundary: Upper Boundary of Gini based on bootstrapped error ranges with a 95% confidence interval.

Plazuela Surface Area (m^2): Gini coefficient for the surface area data based on plazuelas within each nested level and Lower and Upper confidence interval boundaries (see above).

Plazuela Volume (m^3): Gini coefficient for the volume data based on plazuelas within each nested level and Lower and Upper confidence interval boundaries (see above).

Sample Size**:** Number of plazuelas in the sample used for the Gini coefficient. For example, there are 14 plazuelas in Uxbenká District 1. Samples with a * indicate the number of plazuelas that surface area and volume data that were available due to boundaries of the lidar. For example, Ix Kuku'il District 1 has 24 plazuelas, two of which are north of the lidar zone and therefore only 22 plazuelas had surface area of volume calculated.

Time Period that Plazuelas Date to: The number of plazuelas that were occupied during each time period are indicated by the value in the cell. lf = Late Formative, ec 1 = Early Classic 1, ec 2 = Early Classic 2, lc = Late Classic, tc = Terminal Classic. Corresponding dates to the binned time periods are in the table.
