# Supplementary material for: Assessing Classic Maya multi-scalar household inequality in southern Belize
Source: PLoS One. 2021 Mar 24;16(3):e0248169. doi: 10.1371/journal.pone.0248169 (PMC7990175; doi:10.1371/journal.pone.0248169)
Supplement: S1 Text — (PDF) [file pone.0248169.s003.pdf]

S1 Text: Metadata for S1 Table

Polity: Polity name: Uxbenká or Ix Kuku'il.

***Settlement Group*:** Residential group designated during pedestrian survey. Settlement groups consist of one or more plazuelas. Uxbenká contains 136 settlement groups and Ix Kuku'il contains 99 settlement groups.

Neighborhood: Neighborhood designation based on local geography and an Average Nearest Neighbor analysis (Prufer and Thompson 2016; Prufer et al. 2017; Thompson et al. 2018; Thompson and Prufer 2021). Uxbenká contains 20 neighborhoods and Ix Kuku'il contains 16 neighborhoods. "-" means the settlement group is not designated to a neighborhood.

District: Neighborhood designation based on local geography and a directional distribution analysis(Prufer and Thompson 2016; Prufer et al. 2017; Thompson et al. 2018; Thompson and Prufer 2021). Uxbenká contains 3 districts and Ix Kuku'il contains 5 districts. "-" means the settlement group is not designated to a district.

No_of_Strs: Number of structures in each settlement group. "n/a" means that structures were not mapped during pedestrian survey.

Plazuela: Plazuela designation (A, B, C, etc.) based on topography and architectural arrangements of settlement groups. Settlement groups with only one plazuela do not have plazuela designations. Structures within each plazuela are identified in the Str # column. Uxbenká contains 180 plazuelas and Ix Kuku'il contains 122 plazuelas.

Str #: Structure number**.**

Basal area of entire plazuela (m^2): calculated by polygon using the calculate geometry tool in ArcMap 10.7.

Surface area of entire plazuela (m^2): calculated using a lidar derived TIN (see methods section).

Volume of entire plazuela (m^3): calculated using a lidar derived TIN (see methods section).

Time Period: Temporal occupation of plazuelas. "1" = ceramic or AMS ^{14}C date for the time period. lf = Late Formative, ec 1 = Early Classic 1, ec 2 = Early Classic 2, lc = Late Classic, tc = Terminal Classic. Corresponding dates to the binned time periods are in the table.

References

Prufer KM, Thompson AE. Settlement History at Uxbenka: Dispersal and Growth in a Regional Polity. Research Reports in Belizean Archaeology. 2014;11:281–9.

Prufer KM, Thompson AE, Meredith CR, Jordan JM, Ebert CE, Culleton BJ, et al. The classic period Maya transitions from an ideal free to ideal despotic settlement system at the middle-level polity of Uxbenká. J Anthropol Archaeol. 2017;45:53–68.

Thompson AE, Meredith CR, Prufer KM. Comparing geostatistical analyses for the identification of neighborhoods, districts, and social communities in archaeological contexts: A case study from two ancient Maya centers in southern Belize. Journal of Archaeological Science. 2018;97:1–13.

Thompson AE, Prufer KM. Household Inequality, Community Formation, and Land Tenure in Classic Period Lowland Maya Society. J Archaeol Method Theory. 2021 Jan 8;
